# Emerging Integrating Approach to Sensors, Digital Signal Processing, Communication Systems, and Artificial Intelligence

**DOI:** 10.3390/s26072239

**Published:** 2026-04-04

**Authors:** Aleš Procházka, Oldřich Vyšata, Hana Charvátová, Petr Dytrych, Daniela Janáková, Vladimír Mařík

**Affiliations:** 1 Department of Mathematics, Informatics and Cybernetics, University of Chemistry and Technology in Prague, 160 00 Prague, Czech Republic; 2 Czech Institute of Informatics, Robotics and Cybernetics, Czech Technical University in Prague, 160 00 Prague, Czech Republic; vladimir.marik@cvut.cz; 3 Department of Neurology, Faculty of Medicine, Charles University in Hradec Králové, 500 05 Hradec Králové, Czech Republic; 4 Faculty of Applied Informatics, Tomas Bata University in Zlín, 760 01 Zlín, Czech Republic; charvatova@utb.cz; 5 Department of Surgery, 1st Faculty of Medicine & VFN Hospital, Charles University in Prague, 121 08 Prague, Czech Republic; petr.dytrych@lf1.cuni.cz; 6 Department of Sports Medicine, 2nd Faculty of Medicine & FN Motol, Charles University in Prague, 150 00 Prague, Czech Republic; daniela.janakova@fnmotol.cz

**Keywords:** sensor technologies, wearables, digital signal processing, communication systems, artificial intelligence, machine learning, computational methods, biomedicine, motion analysis, neurology, mathematical modeling, renewable energy

## Abstract

Digital signal processing (DSP) methods and artificial intelligence (AI) serve as a unifying platform across diverse research areas and educational courses based on analysis of signals acquired by appropriate sensors and their time-synchronized systems. Autonomous sensor systems having their own batteries, memories, and possibilities of wireless communication form the core of modern technological systems. The interconnection of sensors for data acquisition, methods for advanced analysis of signal features, and collaborative evaluation promotes both theoretical learning and practical problem solving in professional practice. This paper emphasizes a common mathematical foundation for the processing of data acquired by different sensor systems, and it presents the integration of DSP and AI, enabling the use of similar theoretical methods in different applications, including robotics, digital twins, neurology, augmented reality, and energy optimization. Through selected case studies, it shows how a combination of sensor technology for data acquisition and the use of similar computational methods, visualization, and real-world case studies strengthens interdisciplinary collaboration. Findings of this paper demonstrate how integrating AI with DSP supports innovative research and teaching strategies, redefines the field’s educational role in the digital era, and points to the development of new digital technologies.

## 1. Introduction

Sensor technologies, digital signal processing (DSP), communication systems, and artificial intelligence (AI) methods provide a broad interdisciplinary platform with applications across numerous research fields [1,2,3,4,5], including healthcare monitoring, rehabilitation [6], environmental sensing, and industrial automation. Present sensor networks can provide continuous time-series data and sequences of images that motivate a fast development of signal processing methods, AI, and machine learning models [7,8,9,10,11]. Acquired signals can be then used for the detection of neurological problems [12], for the structural health monitoring [13], and for the control of robotic systems [14,15] among others. The progress in sensing technologies for the detection of various physical and environmental conditions and communication systems [16,17] motivates close interdisciplinary collaboration.

The strength of computational methods and sensor technologies lies in bridging differentiated scientific domains, a challenge already discussed in the 17th century. Gottfried Wilhelm Leibniz [18] made profound contributions not only to mathematics and philosophy but also to the idea of interdisciplinary thinking, centuries before it became a central academic goal. He highlighted the danger of excessive specialization, which can make it difficult for scientists from different fields to communicate. Today, the integration of AI and DSP is not only beneficial but transformative, enabling advanced methods in pattern recognition, automatic classification, prediction, and anomaly detection. Adaptive and intelligent systems built on these methods are scalable to large multi-sensor environments and support applications ranging from real-time decision-making to medical monitoring systems.

Numerical methods, digital signal processing, and computational intelligence form a unifying theoretical framework that should directly influence modern scientific areas and educational practices. The mathematical analysis of multichannel and multivariable signals [19] permeates all levels of science and education [20,21,22]. Although DSP applications span vastly different domains—including engineering, technology, and biomedicine—they share a common mathematical foundation. By integrating AI, traditional computational models, and advanced machine learning algorithms into biomedical and engineering signal analysis, it is possible to enhance signal processing methodologies [21,23], particularly in managing complex datasets efficiently.

**Figure 1:**
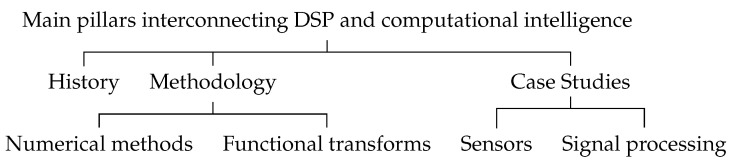
Integration areas of sensors, digital signal processing, and artificial intelligence.

Contemporary research and teaching activities associated with DSP and AI [1,24,25,26] are structured around three main pillars, illustrated in Figure 1, including (i) history: philosophical, scientific, and ethical perspectives; (ii) methodology: computational methods, functional transforms, visualization techniques, software libraries, educational tools; and (iii) case studies: integration of sensor technology and data processing across diverse applications.

The novelty of the contribution is the unifying system-level framework [27] with the review of a core methodology, links to selected papers, and specific case studies. Unlike conventional digital signal processing and artificial intelligence workflows that are typically developed for isolated applications or driven by task-specific learning models, this work introduces a unified interdisciplinary framework that integrates sensor technologies, DSP, and AI through a shared mathematical foundation.

The proposed approach emphasizes domain-independent signal representations, common preprocessing and transform-based feature extraction, and AI-assisted modeling that can be consistently applied across heterogeneous data modalities, including time-series signals, images, and multidimensional sensor arrays. By explicitly coupling sensor system design, signal processing methodology, artificial intelligence, visualization, and domain validation within a single system-level architecture, this paper demonstrates how DSP serves as a unifying core for research, education, and real-world deployment. This integration enables methodological reuse across diverse fields, such as biomedicine, motion analysis, renewable energy, robotics, and augmented reality, while simultaneously supporting innovative research and teaching strategies and interdisciplinary collaboration in the digital era.

The following parts of this paper include Section 1 with notes on the historical and philosophical interconnection of sensors, DSP, and AI, Section 2 presenting common mathematical methods, Section 3 devoted to their use in different applications, and Section 4, Section 5 and Section 6 discussing the integration role of DSP methodology in different applications.

### 1.1. Historical and Philosophical Context of AI and DSP

The intellectual foundations of artificial intelligence (AI) and digital signal processing (DSP) are deeply intertwined with centuries of philosophical and mathematical inquiry. As early as the 13th century, Thomas Aquinas emphasized the importance of reasoning, ethics, and epistemology, themes that remain relevant to the societal impact of AI. Later, Gottfried Wilhelm Leibniz (1646–1716) advanced both mathematics and interdisciplinary thinking, highlighting the dangers of excessive specialization and anticipating ideas central to computation and knowledge representation [1,18,28].

The mathematical framework underlying sensor technology, DSP, and image analysis rests on the work of pioneers such as Isaac Newton, Carl Friedrich Gauss, Jean-Baptiste Joseph Fourier, and Thomas Bayes, whose contributions to numerical methods, probability, and harmonic analysis remain central. The link between spatial and frequency domains, first formalized by Marc-Antoine Parseval, was expanded through modern developments in statistics and signal representation by Johann Karl August Radon, Peter Rayner, Bill Fitzgerald, Ingrid Daubechies, and Nick Kingsbury. These foundations enable today’s computational intelligence methods applied in motion analysis, robotics, biomedical monitoring, human–machine interaction, and large-scale sensor networks.

Modern philosophical trends combined with discussions about the future development of artificial intelligence emerge as the result of a long intellectual trajectory. The lifelong learning shaped by Jan Amos Comenius (1592–1670) resonates strongly with contemporary AI-driven adaptive learning systems. Similarly, literary contributions, such as Karel Čapek’s 1920 play R.U.R., introduced the concept of the “robot” and provoked lasting ethical and philosophical debates about autonomy and human–machine relations.

Gottfried Wilhelm Leibniz, George Boole, and Kurt Gödel laid the groundwork for formal reasoning, while Alan Turing defined computation through the Universal Turing Machine [29]. Today’s sophisticated sensor systems and large-scale mathematical models based on artificial intelligence methods [10,30,31] and extensive databases, including GPT (OpenAI) and BERT (Google), represent a culmination of these traditions, integrating advanced digital signal processing methods with machine learning for classification, prediction, and decision-making. At the same time, they revive enduring questions about the societal role of intelligent machines and their potential risks.

### 1.2. Integration of Sensor Technology, DSP, and Computational Intelligence

Incorporating artificial intelligence into digital signal processing contributes to understanding of how contemporary signal processing techniques are applied to complex challenges in communications, biomedicine, and sensor networks. Both research and courses focused on sensor systems are based on their own datasets processing, remote recording of signals, storage, and analysis using AI tools [32,33,34]. While diverse software packages are available, a solid foundation in the underlying mathematics remains indispensable.

Sensor systems provide data for system analysis and modeling. A very fast technological progress enables the use of complex and sophisticated systems, including magnetic resonance devices, and wearable sensors like accelerometers, gyrometers, RGB, depth, and thermal cameras. Specific data are acquired by robotic systems, drones, and satellites as well. In all these cases, vectors, matrices, and multidimensional bodies of data are recorded, and similar mathematical methods for their processing can be applied in many cases. These steps integrate the sensor system with methods of digital signal processing and computational intelligence tools.

The interconnection of mathematics, computational methods, and advanced visualization provides a compelling foundation for signal processing education as well. By integrating three-dimensional modeling, augmented reality, and interactive video presentations, abstract mathematical concepts become more accessible and engaging. These approaches not only enhance comprehension but also stimulate long-term interest, positioning sensor technologies, DSP, and AI as central pillars of modern interdisciplinary education and research tools.

## 2. Methodology

Interdisciplinary research and modern courses in multidimensional and multichannel digital signal processing [35,36], now increasingly integrated with artificial intelligence, are closely tied to case studies involving data acquisition from wearable sensors [37], wireless communication technologies, and advanced data processing methods. This approach incorporates remote data storage, AI-based analysis [38], and visualization of results through interactive web systems and graphical user interfaces (GUIs). Intelligent signal processing forms the platform for integration of signal processing methods, artificial intelligence, and machine learning, including applications in biomedical diagnostics, engineering, autonomous systems, robotics, and communications [27,39].

Fundamental research blocks associated with data processing and information technologies are summarized in Algorithm 1. While data acquisition and verification methods are closely related to specific physical systems, general tools of signal processing are very close for different problems associated with data processing. Modern computational methods are bridging traditional signal processing methods with modern AI applications [22,40]. These common mathematical and computational intelligence methods are often independent of the data source. Final verification and application are then carried out by domain experts in engineering, biomedicine, or robotics.

DSP provides in this way a unifying mathematical framework that supports a wide range of applications. Sensor systems can record diverse physical, engineering, and biomedical signals ${\left\{s\left(n\right)\right\}}_{n=0}^{N-1}$ and multidimensional arrays with selected sampling frequency $f_{s}$, which often require similar processing pipelines despite their different origins. Seamless integration of sensor technologies, AI concepts, and DSP methods into research [33] interconnects theoretical knowledge and practical experience.

Mathematical processing of signals, images, and multidimensional objects recorded by separate sensors is often based on the use of numerical methods and (partial) differential equations describing models of real systems. Owing to possible errors in observed multidimensional and multichannel sequences, digital filters are often used for data denoising and resampling. All cases studies were evaluated by Matlab version 2025a (Mathworks, Natick, MA, USA) and Comsol Multiphysics version 6.3 (COMSOL, Stockholm, Sweden) software.

Functional transforms are another general research area. Both signal analysis in the frequency domain using discrete Fourier transform and Wavelet decomposition form another general research area. Features evaluated either in the frequency or scale domains form the basis for classification and prediction tasks.
**Algorithm 1** Data acquisition, general tools of signal processing, and implementation**Data acquisition:** design of sensor technologies for data acquisition, proposal of communication links for information transmission, and data storage systems.**General tools of signal processing:** DSP and AI methods including:Multidimensional and multichannel signal preprocessing, including initial error rejection, resampling by approximation and interpolation methods, time synchronization, data registration [41] for long term evaluation of system changes, and digital filtering for noise rejection.Functional transforms [42,43] ensuring scale-, shift-, and rotation-invariant detection of data components and extraction of signal features based on Radon, discrete Fourier, wavelet, and z-transforms in many cases.Data analysis including extraction of signal features in time and functional domains for recognition, prediction, and classification of signal components.Mathematical modelling of real systems using AI tools, computational algorithms, and machine learning methods for real systems analysis, monitoring, and control.3.**Verification:** validation of results, and their use in real situations.

Additional advanced methods include adaptive signal processing tools, general machine learning methods, and deep learning algorithms used in specific research areas including object recognition and control of robotic systems. But, even in these complex problems, we find a common background based on data analysis and processing of very extensive database systems.

## 3. Case Studies

The following case studies present selected applications of sensor use for data acquisition. They illustrate the interdisciplinary role of signal processing, demonstrating how similar methods can be applied to process multidimensional and multichannel signals acquired across diverse domains [1,44].

Studies that combine advanced sensor systems, mathematics, and artificial intelligence provide a highly engaging foundation for students of signal processing. Selected case studies further motivate the exploration of complex computational methods.

### 3.1. Data Processing in Neurology, Stomatology, and Surgery

Electroencephalography (EEG) represents one of the most important methods for non-invasively monitoring brain activity and studies of neural dynamics [45]. The integration of artificial intelligence, digital signal processing, and machine learning, including deep learning, allows very fast progress in this area.

**Figure 2:**
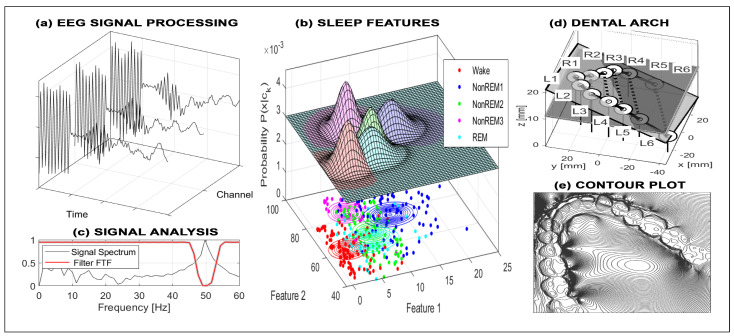
Biomedical data analysis presenting (**a**) EEG signal denoising, (**b**) sleep-stage classification showing distribution probabilities across five classes using mean power in two frequency bands for 30 s segments, (**c**) EEG signal analysis, (**d**) dental arch registration, and (**e**) dental body contour plot.

EEG signals acquired according to Figure 2a stand for potentials recorded by electrodes in specific locations on the head, and they form a matrix, with each row associated with one sensor. Figure 2a–c illustrate EEG signal denoising, analysis, and sleep-stage detection [46,47] based on overnight recordings sampled at 200 Hz. Classification into five sleep stages was performed using Bayesian methods applied to features defined as the mean power within two frequency bands, evaluated over 30 s segments.

Another important biomedical technology is based on 3D scanning, which provides multidimensional arrays important for analysis in different biomedical areas, including surgery and stomatology. Figure 2d,e demonstrate the application of such images in stomatology using intraoral scanning technology [48]. A three-dimensional mathematical model of the dental arch enables detailed digital analysis during treatment of dental disorders and supports 3D printing of replacement components. Methods based on image registration are also critical in surgery [49], where they assist in detecting specific anatomical components and monitoring treatment progress.

Sensors used in diffuse reflectance spectroscopy provide matrices enabling the finding of features for early detection and classification of dental caries using machine learning and deep learning approaches. Specific studies [50] are devoted to machine learning for the classification and regression of diffuse reflectance spectroscopy signals with a strong potential for tissue differentiation in clinical practice.

The applications of DSP in biomedicine, neurology, and surgery [51] are broad, motivating studies of sensor systems, communication technologies, and advanced general computational methods [52]. General computational intelligence techniques are employed for image enhancement, digital filtering, and feature extraction.

### 3.2. Motion Features Estimation and Classification

Accelerometric and gyrometric sensors, heart rate monitors, and global navigation satellite systems (GNSSs) for positioning detection provide valuable data for gait analysis, motion feature estimation, and the detection of neurological disorders [53]. Data can be recorded either by specific sensors or sensors embedded in mobile phones, with AI-based tools supporting the evaluation and visualization of results.

**Figure 3:**
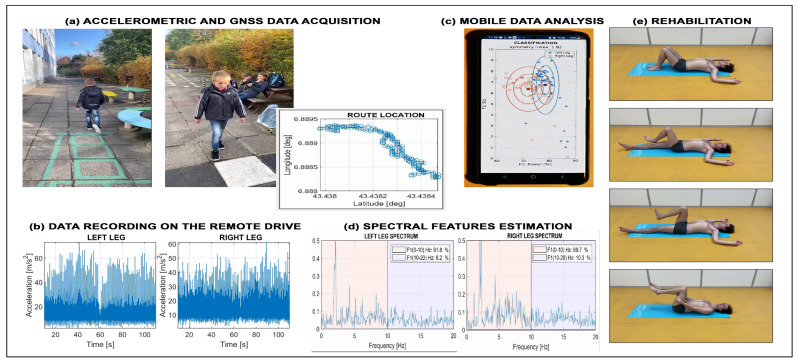
Use of wearables for motion data acquisition presenting (**a**) the use of sensors for accelerometric and GNSS data acquisition during walking tests, (**b**) accelerometric data recorded on the left and right legs, (**c**) mobile Matlab use for data processing using the remote drive and specifying cluster centers with multiples of standard deviations, (**d**) detailed analysis of spectral gait features for evaluation of the gait symmetry, and (**e**) use of accelerometric data acquisition in rehabilitation.

Figure 3a–d show an example of walking pattern analysis in children with motion disorders [54,55]. After initializing the sensor system and storing datasets in a remote drive, spectral features of accelerometric data were used for symmetry coefficients estimation by the remote computational Matlab system. Such projects are engaging both for the children under examination and for neurologists performing the motion treatment.

Motion sensors are equally important for rehabilitation studies. Figure 3e illustrates accelerometric data acquisition during rehabilitation exercises. Analysis of these signals supports the design of pre-surgery training programs aimed at reducing the risk of postoperative complications. These applications foster interdisciplinary collaboration, encourage the creation of video-based training systems, and allow comparison between real and ideal motion patterns for each individual. Spectral analysis of accelerometric data provides additional insight by quantifying the frequency components of signals from the left and right sides of the body to assess motion symmetry.

Wearable motion sensors motivate studies of principles of accelerometric data acquisition, the mathematics of spectral analysis, and the integration of AI tools for automated evaluation through computational web platforms.

### 3.3. Physical Activities Recognition

Digital signal processing methods and artificial intelligence provide powerful tools for motion recognition, physical activity evaluation [56], and monitoring of sport activities. The use of wearable sensors in this area represents an attractive direction pointing to the integration role of different research areas. Associated problems typically use global navigation satellite systems (GNSSs) for position tracking [57] with additional biomedical sensors measuring variables such as heart rate and blood oxygen concentration.

**Figure 4:**
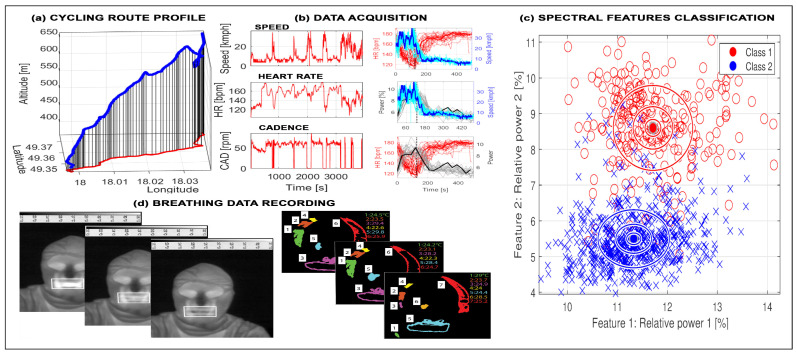
Signals recorded during a cycling experiment presenting (**a**) cycling route profile, (**b**) time series of speed, heart rate, and cadence, (**c**) spectral features classification with cluster centers and multiples of standard deviations, and (**d**) breathing frequency detection using thermal imaging.

Examples of motion detection are presented in Figure 4a–c that show a cycling route profile, biomedical features, and classification results linked to route conditions [58]. Such analyses provide cyclists with feedback on fitness level and performance using AI tools. The sensor set can be extended with a thermal camera to detect breathing frequency, as shown in Figure 4d, where adaptive mouth-area detection enables estimation of temperature fluctuations and their periodicity.

**Figure 5:**
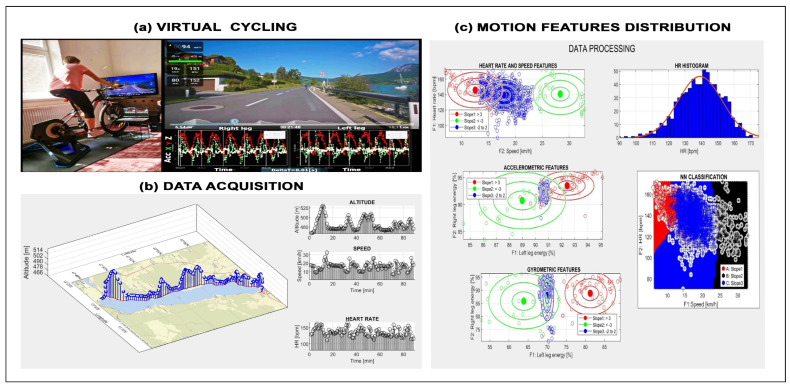
Virtual cycling data presenting (**a**) principle of virtual cycling, (**b**) mapping environment and selected acquired signals, and (**c**) motion features classified across route segments with different slopes.

Figure 5 illustrates the concept of virtual cycling [59,60,61], where a route is defined in a mapping environment and sensor data are collected during simulated tours. Motion features are then classified across route segments with different slopes, as shown in Figure 5c.

**Figure 6:**
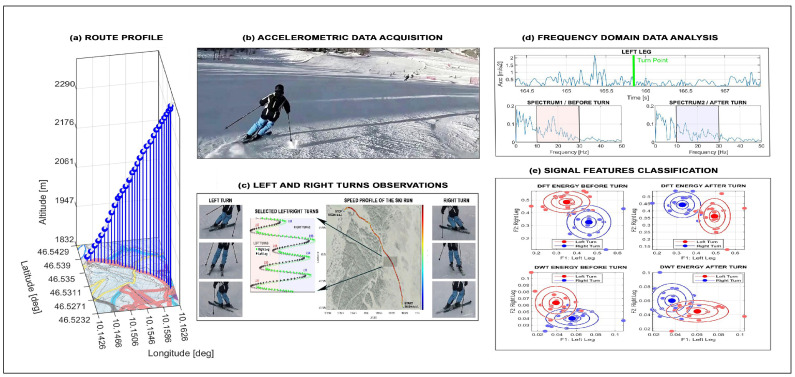
Accelerometric and gyrometric data recorded during downhill skiing presenting (**a**) route profile, (**b**,**c**) motion data acquisition, (**d**) frequency domain analysis, and (**e**) classification of left and right turns in frequency and scale domains.

Sports activities such as running, skiing, and cycling also combine GNSSs with biomedical signal acquisition using the set of selected sensors. Figure 6 shows accelerometric and gyrometric data from downhill skiing [62,63,64], including route profile, raw motion signals, frequency-domain analysis, and classification of left versus right turns using frequency and scale-domain features.

These case studies highlight the role of GNSSs and wearable sensors in analyzing a wide range of physical activities, from walking and running to skiing, integrating sensor technologies with general signal processing methods. They also motivate the study of satellite navigation, synchronization of multimodal sensor data, and appropriate choices of the sampling frequency.

### 3.4. Optimization of Renewable Energy Sources

**Figure 7:**
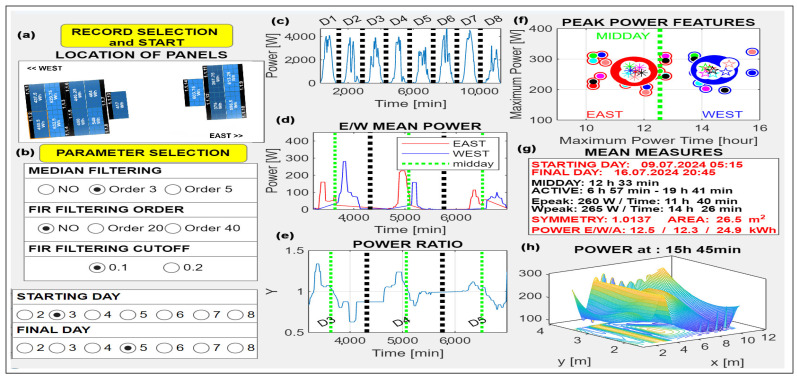
Graphical user interface for power data processing in an east/west-oriented PV system presenting (**a**) data import and panel layout, (**b**) parameter selection, (**c**) total power of a selected string, (**d**) mean power of east- and west-oriented panels, (**e**) normalized east/west power ratio, (**f**) peak power features, (**g**) numerical results, and (**h**) 3D power distribution at a selected time.

An attractive application of different sensor systems is in the use of digital signal processing and artificial intelligence methods in the analysis, optimization, and fault detection of photovoltaic (PV) systems [65,66,67,68] as presented in Figure 7. Machine learning, artificial intelligence techniques, and algorithms are used for the proposal of new methods for complex design and forecasting of PV plants and their maintenance [69,70]. Temperature and other sensors can monitor the health of battery systems, and drones equipped by RGB cameras and thermal sensors are used to detect disorders of PV panels.

Figure 7 shows the graphical user interface of an east/west-oriented PV system designed for analysis of remotely recorded panel data. Signal processing tasks include linear and nonlinear filtering, feature extraction, and classification of performance patterns. Thermal imaging combined with AI is also valuable for detecting PV panel defects. These results motivate the study of DSP methods, AI tools, the physical principles of irradiance, and aspects of material engineering.

Sensor systems and AI can further contribute to renewable energy generation by optimizing solar panel orientation, supporting robotic systems for automated cleaning, and enabling efficient power control.

### 3.5. Thermal Systems Modeling and Heat Control

Thermal sensors and image processing provide an important domain for the integration of DSP and AI methods with applications in thermal engineering, modeling, prediction, and optimization [71,72] to increase energy efficiency, system reliability, and to improve environmental sustainability.

Projects involving computational modeling of heating systems and building insulation are often related to system design in COMSOL or similar platforms. The deep knowledge of material engineering requires verification of models by digital data from thermal cameras acquired from a real system.

**Figure 8:**
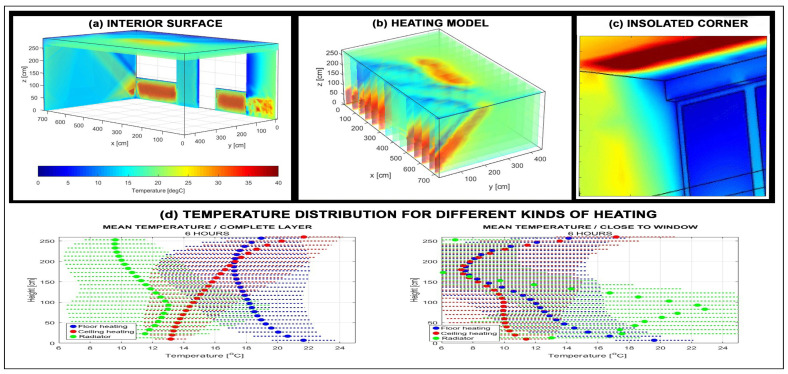
The 3D model of temperature distribution in a thermally insulated room presenting (**a**) temperature distribution in the walls, (**b**) air temperature in the spatial model, (**c**) temperature field around a critical room corner, and (**d**) distribution in selected parts of the room with different heating sources.

Figure 8 illustrates a 3D COMSOL model of temperature distribution in a thermally insulated room [73,74,75,76]. The simulation results were validated using thermal camera measurements, which provided temperature maps of critical areas such as walls, room corners, and zones influenced by different heating sources.

The use of thermal sensors requires a thorough understanding of thermal noise and the challenges associated with their calibration. Related projects highlight the interdisciplinary integration of DSP, AI, and material science, offering students practical experience in modeling, measurement, and optimization of renewable and thermal energy systems.

### 3.6. Summary

**Figure 9:**
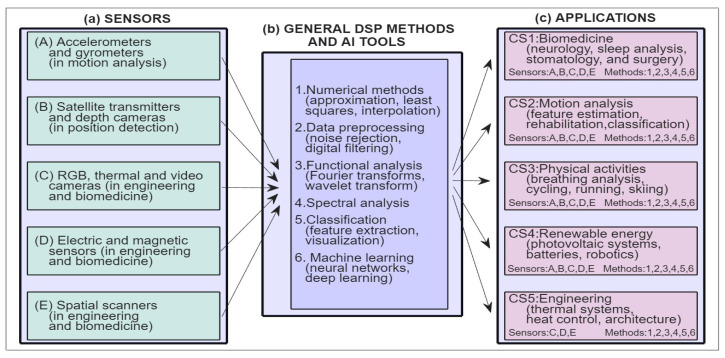
Compact overview of information engineering in data processing presenting (**a**) summary of different sensors used for digital data acquisition, (**b**) unifying computational core of general methods for multidimensional signal processing, and (**c**) specific implementations of data processing in different application areas.

Case studies point to the close interconnection among different sensor systems, data acquisition, and common mathematical methods for processing the resulting multidimensional measurement matrices. Figure 9 presents a summary of selected sensors and their applications in data analysis.

The resulting signals, recorded as sampled values, can then be processed using similar mathematical methods. Although the mathematical core is similar, the implementation of the results is application-specific and related to scientific research, practical needs, biomedical diagnostics, rehabilitation, advances in technological systems, robotics, satellite communications, and engineering design, among other areas.

## 4. Discussion

Results point to common methods based on the processing of vectors and matrices recorded by wearable sensors and sensor systems in different applications. Accelerometers and global navigation satellite systems sensors find their use in neurology, rehabilitation, fitness monitoring, and specific engineering systems. RGB, depth, and thermal cameras are important in all these applications and moreover in biomedicine, surgery, stomatology, and photovoltaics.

Common methodology based on information processing algorithms includes numerical methods, linear and non-linear digital filters, functional transforms (including Radon, discrete Fourier, and wavelet transforms), and methods of computational intelligence. Most of these methods are based on multidimensional and multichannel signal processing, which is not dependent on specific applications, and can form the general evaluation core for many different applications.

Similar mathematical methods for the evaluation of digital signals enable close collaboration of specialists in different areas. This approach is supported by the rapid technological progress of sensor systems for data acquisition and specific tools for the implementation of computational results in the real-world environment including robotic systems and visualization tools.

The widespread adoption of computational intelligence in signal processing applications highlights the growing need for interdisciplinary collaboration, as well as critical reflection on emerging ethical challenges and philosophical questions concerning the role of individuals in a technology-driven world. The unifying role of information engineering and signal processing provides a shared framework for scientists across diverse fields. The integration of computational methods with advanced visualization, three-dimensional modeling, and augmented reality represents a rapidly evolving area of both research and education.

The present paper contributes a formal, reusable, and domain-diagnostic framework for integrating sensor technologies, digital signal processing, and artificial intelligence. The framework is grounded in shared mathematical signal representations and defines a modular processing architecture that can be consistently applied across time-series, image-based, and multidimensional sensor data. Research areas are closely interconnected with educational courses that combine computational intelligence with case studies often using online platforms for data sharing, synchronization, and real-time analysis of data accessible from mobile devices. Experiments involving signals recorded on smartphones promote self-directed learning of analysis methods and provide an accessible alternative to processing professionally recorded signals from engineering laboratories, satellite systems, or clinical environments.

This paper presents a review of selected papers and ideas unifying a methodological approach to sensor systems, digital signal processing, communication links, and artificial intelligence tools with selected case studies. It contributes to the multidisciplinary approach to these topics and a unifying core, based on information processing, common for most applications.

## 5. Conclusions

Digital signal processing has gained increasing significance in recent years, owing to its unifying role in providing a common set of methodologies based on shared mathematical principles and its central importance in information engineering across multidisciplinary domains. History of signal processing methods is based upon research of many philosophers and mathematicians in last ages with a fast growth that started in the middle of the last century, supported by the fast evolution of engineering and communication technologies and increasing availability of digital computers [77,78].

The combination of signal acquisition tools, sensors, communication links, and AI methods motivates further progress and innovative research in many areas. Signal processing forms a common interdisciplinary backbone across sensors, communication systems, digital signal processing, and artificial intelligence. All these fields deal with signals: acquiring them, transmitting them, transforming them, and extracting information from them.

Unlike conventional DSP and AI pipelines that are optimized for single applications or algorithms, this work introduces a unified, sensor-to-AI interdisciplinary framework grounded in shared mathematical principles, enabling reusable workflows across biomedical, motion, energy, robotics, and educational domains. Motion sensors, visual sensor systems, and intelligent optical fiber sensors [79,80,81] form an important subcategory of sensors important for many present applications.

## 6. Future Directions

Future directions of sensors and computational intelligence include interdisciplinary collaboration in many areas. Demands for high precision and high speed in machine vision systems belong to one of them. Information forecasting [82] forms another important area across various fields of engineering, motion monitoring, and medical diagnosis. Digital twins are becoming very important for the verification of mathematical models in real situations for state estimation, prediction, and control. A development of common mathematical background for different applications and general computational methods forms an important research and educational area [1] for the efficient progress of information engineering. Quantitative comparisons with domain-specific state-of-the-art methods form another direction of future research.

General DSP techniques and machine learning methods follow the technological progress of data acquisition and extensive use of database systems, supported by AI, and using similar intelligent computational tools. The interconnection of these areas has the potential to serve as an efficient interdisciplinary platform that not only advances research and education but also adapts to emerging technologies.

## Data Availability

Datasets and video abstract associated with separate case studies are stored at the IEEE DataPort (https://ieee-dataport.org/ (accessed on 30 March 2026)), Zenodo repository (DOI: 10.5281/zenodo.19236646), and specified in detailed papers cited in individual sections.
